# Health Effects of PCBs in Residences and Schools (HESPERUS): PCB – health Cohort Profile

**DOI:** 10.1038/srep24571

**Published:** 2016-04-19

**Authors:** Elvira Vaclavik Bräuner, Zorana Jovanovic Andersen, Marie Frederiksen, Ina Olmer Specht, Karin Sørig Hougaard, Niels Ebbehøj, Janice Bailey, Aleksander Giwercman, Kyle Steenland, Matthew Paul Longnecker, Jens Peter Bonde

**Affiliations:** 1 Research Center of Prevention and Health, Center of Health, Capital region of Denmark, Rigshospital - Glostrup, Copenhagen University, Denmark; 2 Department of Occupational and Environmental Medicine, Bispebjerg - Frederiksberg Hospital, Institute of Public Health, University of Copenhagen, Copenhagen, Denmark; 3 Department of Public Health, Center for Epidemiology and Screening, Faculty of Health Sciences, University of Copenhagen, Denmark; 4 Department of Energy, Environment and Indoor Climate, Danish Building Research Institute, Aalborg University, Denmark; 5 National Research Centre for the Working Environment, Copenhagen, Denmark; 6 Department of Animal Sciences, Centre de Recherche en Biologie de la Reproduction, Université Laval, Canada; 7 Molecular Reproductive Medicine, Department of Translational Medicine, Lund University, Sweden; 8 Department of Environmental Health, Rollins School of Public Health, Emory University, USA; 9 Epidemiology Branch, National Institute of Environmental Health Sciences, NIH/DHHS, Research Triangle Park, NC, USA

## Abstract

Polychlorinated-biphenyls (PCBs) were introduced in the late 1920s and used until the 1970s when they were banned in most countries due to evidence of environmental build-up and possible adverse health effects. However they still persist in the environment, indoors and in humans. Indoor air in contaminated buildings may confer airborne exposure markedly above background regional PCB levels. To date, no epidemiological studies have assessed the health effects from exposure to semi-volatile PCBs in the indoor environment. Indoor air PCBs are generally less chlorinated than PCBs that are absorbed via the diet, or via past occupational exposure; therefore their health effects require separate risk assessment. Two separate cohorts of individuals who have either attended schools (n = 66,769; 26% exposed) or lived in apartment buildings (n = 37,185; 19% exposed), where indoor air PCB concentrations have been measured were created. An individual estimate of long-term airborne PCB exposure was assigned based on measurements. The cohorts will be linked to eight different national data sources on mortality, school records, residential history, socioeconomic status, and chronic disease and reproductive outcomes. The linking of indoor air exposures with health outcomes provides a dataset unprecedented worldwide. We describe a project, called HESPERUS (Health Effects of PCBs in Residences and Schools), which will be the first study of the long term health effects of the lower-chlorinated, semi-volatile PCBs in the indoor environment.

Polychlorinated biphenyls (PCBs) were used worldwide in the 1920s as coolants, dielectrics, and lubricants in transformers, capacitors, and other electrical equipment^1^. Beginning in the 1950s they were used as additives in building materials such as elastic sealants, caulk, grouts and paints, as well as flame retardants in coatings of acoustic ceiling tiles^1^. In the 1970s, PCBs were discontinued in the so called “open applications”, including sealants and paints, due to environmental build-up and potential adverse health effects. They were later discontinued in “closed applications”, such as capacitors and transformers^1^. Due to their stability and resistance to degradation^1^, PCBs are still present within building construction materials in homes and schools and will remain so for centuries to come. In Denmark, 37% of the total housing stock was constructed in the period when PCBs were used in building materials^2^. A recent nationwide mapping of PCB in buildings indicated that 10% of all Danish buildings have materials such as caulking, sealants and paints with more than 5000 ppm PCB, and surveys have shown that PCBs in these buildings may result in indoor air concentrations exceeding 300 ng/m^3 ^2,3, which is the limit above which action to reduce levels is recommended by both the US EPA and the Danish Health and Medicines Authority (Table 1). A similar situation is likely in most industrialized countries. For example, in the US, it is estimated that over 75 million kilograms of PCBs were sold for use as plasticizers in building materials^4^. Thus, a large number of buildings constructed from the 1950s to the 1970s may include PCB-containing materials. Indoor air levels as high as 5000 ng/m^3^ have been reported in the US^5^, and a recent review estimated that as many as 25,920 US public schools are contaminated by PCBs^3^.

There are 209 different PCB congeners with varying chemical and physical properties. Mixtures of congeners have been applied in sealants, caulking and other building materials. The lower-chlorinated congeners that predominate in indoor air, are primarily non dioxin-like (non-planar and not binding to aryl hydrocarbon receptors), and have been much less studied than the higher-chlorinated PCBs present in the diet and in previous occupational exposures. Indoor PCBs are semi-volatile and are slowly, but continuously, released to the air^6^. This is further complicated by re-adsorption to other surfaces, such as painted walls or lacquered floors, leaving a much larger area for re-emission. As a consequence, PCB remediation by removal of original sources or increased ventilation in these buildings does not remove the contamination of the air. Consider as a realistic example a small room contaminated with 500 g of PCB in the caulking and an air change rate of 0.5 h^−1^; it would take 44 years to remove just 1 g of PCB by ventilation^7^. Therefore, PCB levels in indoor air in these environments may be many orders of magnitude higher than outdoor regional air levels or indoor air levels in uncontaminated buildings (typically: 0–10 ng/m^3^), as documented in a number of studies in Europe and US (Table 1). We have recently demonstrated that exposure in contaminated buildings significantly contributes to blood plasma concentrations, particularly of the lower-chlorinated PCB-congeners^8^. As a consequence, PCBs in the indoor environment may contribute up to 63% of the overall PCB exposure in adults and 36% in toddlers^8^,^9^,^10^,^11^,^12^,^13^,^14^,^15^. Altogether, the evidence indicates that the main non-occupational source of exposure to PCBs in the entire general population is airborne, challenging the long-held view identifying diet as the main non-occupational exposure. Airborne exposure to semi-volatile PCBs in the indoor environment requires specific risk assessment, and to date no epidemiological studies have assessed the potential contribution to adverse health effects from this exposure. Recent reviews regarding evidence on the health effects of the higher-chlorinated PCBs usually conferred via the diet shows associations with malignant disease, ischemic heart disease, type II diabetes, thyroid dysfunction, neurotoxicity and reproductive health^1^,^16^,^17^, but this knowledge does not allow for appropriate risk assessment of indoor air exposure to PCBs, with its different profile of PCB congeners. In western societies, we spend most of our time indoors, particularly in homes and schools. Therefore the potential risk of PCBs to the health of current and future generations, particularly in vulnerable periods of life such as the prenatal period, childhood, puberty, and late life, is a prominent public health issue. To date there have been no feasible options available to study the effects of these congeners in human populations.

In this paper we describe the “birth” of two novel Danish cohorts which combine nationwide health and socioeconomic registers with measured and estimated indoor exposure to air PCBs: one of residents in apartments and one of pupils attending public schools. These cohorts can link indoor air exposures with health outcomes providing a dataset unprecedented worldwide, offering insight into the health effects of indoor air PCBs. Here we describe the creation of these cohorts in the project HESPERUS (Health Effects of PCBs in Residences and Schools).

## Who is in the Cohort?

**Figure 1:**
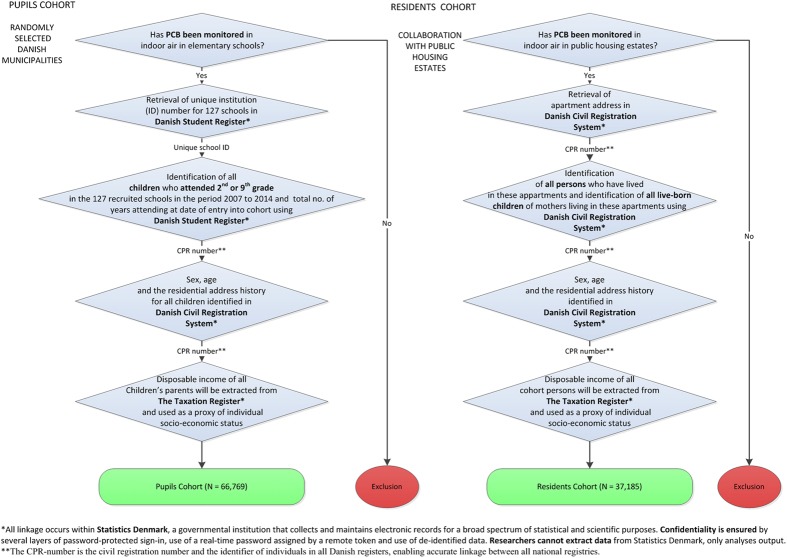
Creation of school children and apartment resident PCB cohorts.

We have created two separate cohorts: pupils in elementary schools, and residents of apartments with measured indoor air PCB concentrations (Fig. 1). Each individual will be assigned an estimated long-term PCB exposure as outlined below.

### What was measured?

PCB measurements were carried out by accredited laboratories. Air samples were collected by stationary, active air samplers placed 1–1.5 meter above floor level and with typically 8–24 hours sampling time equivalent to 1.0–2.5 m^3^ indoor air^6^. At least the six indicator PCB congeners (PCB 28, 52, 101, 138, 153 and 180) were analyzed by gas-chromatography coupled to high resolution mass spectrometry (GC-HRMS)^6^ and the total PCB concentration was estimated as the sum of the 6 indicator congeners multiplied by a correction factor of 5. This correction factor is commonly used to estimate total PCB content in air when the composition of the PCB source is unknown and is an accepted norm amongst all accredited laboratories in Denmark and the US EPA in field sampling (EN15308:2008). Furthermore, it has been incorporated into European and US EPA standards for analysis of PCBs^18^.

#### PCB measurements: data collection

Following extensive media coverage of reports showing unexpectedly high concentrations of PCB congeners in some public buildings and dwellings (including elementary schools), the Danish government launched a national action plan to ameliorate hazards due to indoor exposure of PCBs in 2011^2^. One of 19 actions was a nationwide mapping of PCB content in building materials and measurements of PCB congeners in indoor air in representative samples of buildings including elementary schools and social housing^2^. Measurements were carried out by several independent universities and environmental consultants. All analyses were carried out at accredited laboratories. The nationwide ation is completed but measurements are ongoing, all municipalities are encouraged to measure all schools in their jurisdiction. This mapping and the following independent monitoring campaigns of PCB exposure provided for unique opportunity to use these measured PCB exposure levels for study of health effects, as described in this project. Furthermore, action was taken to bring levels down in the schools or public housing estates in which PCB levels above the Danish recommended action levels for PCB in indoor air were detected. Recommended Danish action levels are as follows: <300 ng/m^3^: exposure not presumed to pose a risk; 300–3000 ng/m^3^: action plan required to bring levels down; >3000 ng/m^3^: immediate need for action to bring levels down.

**Figure 2:**
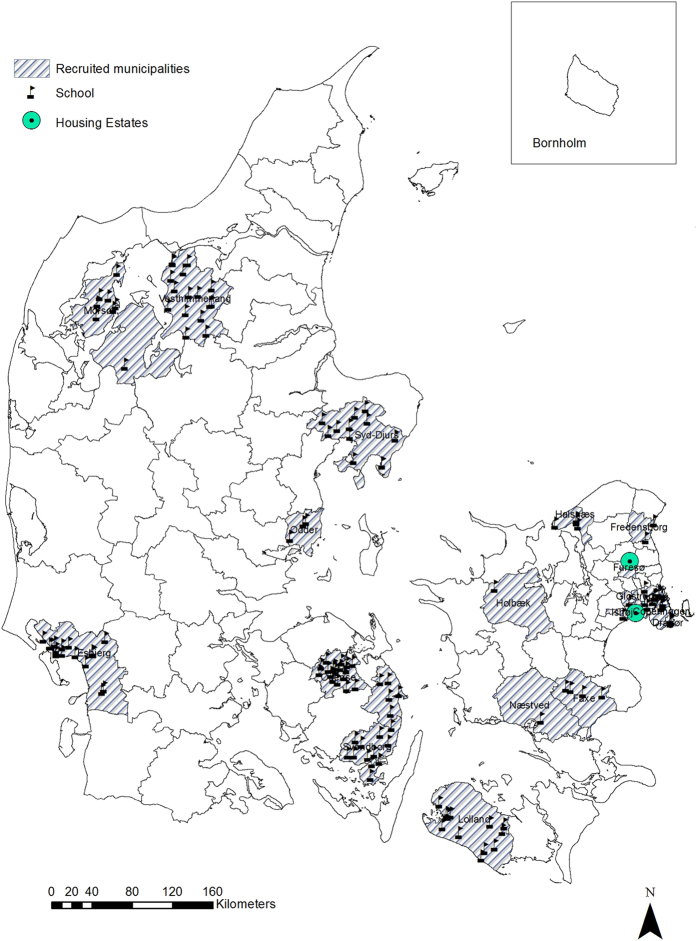
Geographical location of the 127 schools from school children PCB cohort and 2 social housing estates from apartment residents PCB cohort in Denmark. Generated in ArcGIS Desktop: Release 10. Redlands, CA: Environmental Systems Research Institute (ESRI) 2011 (URL: http://www.esri.com/software/arcgis/arcgis-for-desktop).

We identified *elementary schools with measured PCB indoor levels* via contact with 22 municipalities for which we had *a-priori* knowledge that PCBs had been monitored in their schools (Fig. 1). These 22 municipalities were selected randomly among all municipalities where schools had been measured (n = 36), to represent different regions of Denmark. Of the 22 municipalities we contacted, 20 had performed PCB measurements in indoor air in their schools within the period 2010 to 2014 (Fig. 2), whilst 2 only performed measurements in caulking and sealants. We have now collected the PCB measurements in indoor air from all 127 schools that had available PCB measurements within these 20 municipalities (Table 2).

Danish social housing is a non-profit sector that covers the whole country and comprises around 7000 housing estates, 560,000 dwellings and more than 1,000,000 inhabitants, representing almost one fifth of the Danish population. The social housing system in Denmark offers apartments at a reasonable cost for the general population, but is not specifically for low-income or socially disadvantaged families. We identified 2560 *apartments with PCB indoor levels measured by accredited laboratories* located within two housing estates within the social housing sector, via contact with the centralized administration office (Fig. 1).

We selected these two estates based on *a-priori* knowledge obtained from previous monitoring campaigns that had identified the apartment buildings within the housing sector with PCB contamination (Fig. 2). *Farum Midtpunkt* comprises 27 apartment buildings with 1645 individual apartments, of which 297 are contaminated (5^th^, 25^th^, 50^th^, 75^th^ and 95^th^ percentiles: 305, 534, 859, 1261 and 2781 ng/m^3^)^6^. *Brøndby Strand* comprises 12 apartment buildings with a total of 885 individual apartments of which 405 are contaminated 5^th^, 25^th^, 50^th^, 75^th^ and 95^th^ percentiles: 507, 877, 1246, 1875 and 2990 ng/m^3^). Both estates were built during the same time period from 1970–1974, but each include apartment buildings with high as well as low level (no) air PCB contamination (Table 3). This will enable comparisons of high- and low-level exposed populations with balanced socioeconomic status.

### Cohort creation

In Denmark, information on births, deaths, immigration and emigration, disease incidence, education and social conditions has been collected in registers for several decades and high-quality data with complete coverage of the whole population are available for research purposes. In the registers, each citizen is identified by a unique identification number, called a CPR-number (social security number). This number has been given to all citizens of Denmark since the establishment of the Danish Civil Registration System (CRS)^19^ in 1968 and allows accurate linkage between registers. These registers offer great possibilities for population-based epidemiological studies of health effects related to environmental and lifestyle exposures that are not feasible in most other countries. In the present study, these registers were used to create two novel PCB cohorts (Fig. 1).

1. We used the Danish Student Register to identify a *cohort of 66,769 school children* who attended one of the 127 public schools with measured PCB in indoor air, in either 2^nd^ or 9^th^ grade within 2007–2014^20^. The 2^nd^ and 9^th^ grades were selected as these are pertinent to our assessment of cognitive function. Population, gender distribution and years of attendance in school according to exposure level are shown in Table 2, along with their classification by air PCB levels. Twenty-five percent of these schools have PCB levels >300 ng/m^3^, whilst fifty-four percent have PCB air levels between 1–300 ng/m^3^. In 2015, there were 1313 elementary public and 250 private schools in Denmark (pupils aged 6–16 years), thus the cohort of schools represent 10% of all elementary public schools (and 8% of all schools) in Denmark. The duration, calendar period, and level of PCB exposure (ng/m^3^, continuous scale) will be estimated for each identified school child based on PCB measurements undertaken in these schools.
2. We identified a *cohort of 37,185 residents* who live or have lived in the targeted residencies with measured PCB levels in indoor air by linkage of these addresses to the Danish Civil Registration System from 1971 to 2015. Each resident will be assigned an exposure level (ng/m^3^, continuous scale), duration of exposure, and calendar period, based on exposure estimates for their individual apartments. Apartments in contaminated buildings generally have similar PCB levels. The cohort consists of approximately 5,992 residents from apartments with high PCB-levels (>100 ng/m^3^) and a reference group of 31,193 non-exposed cohort members (≤100 ng/m^3^) with comparable socioeconomic status living in non-contaminated apartments in the same geographical areas (Table 3).

Inclusion of residents and pupils into the two cohorts will not be restricted by health or social criteria. However, within each of the specific follow-up studies included in HESPERUS we will exclude individuals who had the disease or adverse health outcomes of interest before the start of follow-up, to facilitate analyses of incident health outcomes. Potential confounding factors such a familial predisposition and socio-economic status will be taken into account based on information that can be retrieved from Danish health registries.

### Data sources - Nationwide Danish Registers Pertinent to Outcomes identified

**Figure 3:**
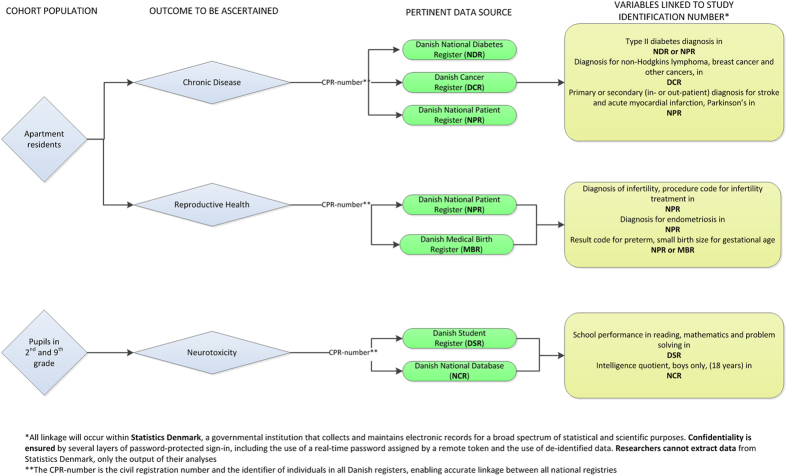
Nationwide registers pertinent to HESPERUS outcomes and variable output.

Eight different national data sources will be employed to gather information on vital status (birth, death, emigration), cognitive function, school attendance, residential address history, socio-economic status as well as health and reproductive outcomes (Fig. 3), including the: Danish Civil Registration System, the Taxation Registry, Danish Conscription Database, Danish Student Register, Danish National Patient Register, Danish National Prescription Registry, Danish National Diabetes Register, Danish Cancer Registry, and Medical Birth Register. All linkage and statistical analyses will occur within Statistics Denmark, a governmental institution that collects and maintains electronic records for a broad spectrum of statistical and scientific purposes. Data will be stored on the Statistics Denmark platform and confidentiality is ensured by several layers of password-protected sign-in including the use of a real-time password assigned by a remote token and the use of de-identified data. Researchers can see and analyse data when logged in but cannot extract data from Statistics Denmark and only the outputs of the analyses are available.

These health outcomes were selected based on previous evidence of health effects associated with the dietary or occupational exposure to higher-chlorinated PCBs. But the cohorts can also be extended to include other pertinent health outcomes for interested researchers.

The *Danish Civil Registration System* was established in 1968 for administrative purposes^19^ and as already described all Danish residents (native or immigrated) are registered and assigned a unique personal identification number, called the CPR-number. The register contains current and historical information on date of birth, death, immigration and emigration, as well as residential address information on all persons living in Denmark. The CPR-number is the identifier of individuals in all Danish registers, enabling accurate linkage between all national registries.

The *Taxation Registry* provides information on disposable income, defined as income for a single family member after taxation and adjustment for the number of family members (http://www.dst.dk/en.aspx). Income will be used as a source of information about socioeconomic status at the individual level.

The *Danish Student Register* (DSR) was established in 1977^20^ and contains unique institution codes that enable identification of all schools in Denmark as well as information on the school performance of children enrolled in these schools. Since 2004, school performance grades were available at all levels from pre-school to graduation.

The *Danish National Conscription Register* (NCR) contains intelligence data on nearly all Danish men over the age of 18. In Denmark, Danish men have had to register with the military board for a physical and mental examination of fitness when turning 18 years old or shortly thereafter. In connection with the registration, all examinees complete an intelligence test. The Boerge-Prien test is a 78-item, 45-min validated intelligence test developed for the Danish draft board and is highly correlated with the full-scale IQ on the Wechsler’s adult intelligence scale^21^. These data are available digitally from 2005 onwards in the *Danish Conscription Register*^22^.

The *Danish National Patient Register* (NPR) contains nationwide clinical data on in-patients admitted to Danish hospitals since 1977, and out-patients since 1994^23^. The register contains dates of admission and discharge (or for out-patients, start and end of treatment), and diagnosis code. The International Classification of Diseases (ICD) 8^th^ revision (ICD-8) was used until 1994, and thereafter ICD-10 (ICD-9 was not implemented in Denmark).

The *Danish National Diabetes Register* (NDR) contains information on the incidence of diabetes in Denmark. The register was established in 1995 by linking information in existing registries^24^,^25^. Inclusion criteria include hospital discharge diagnoses of diabetes in the National Patient Register; podiatry for diabetic patients, five blood glucose measurements within one year, or two blood glucose measurements per year for five consecutive years as registered in the National Health Insurance Register (which contains all services provided by general and specialist practitioners since 1973); or two purchases of insulin or oral glucose-lowering drugs within 6 months as registered in the Danish National Prescription Register.

The *Danish Cancer Register* (DCR) was established in 1943 and contains data on the incidence of malignant neoplasms (and certain precancerous and benign lesions) in the Danish population^26^. Reporting to the DCR has been mandatory since 1987. Since 2004 reporting has been mediated via the NPR, such that when a cancer is entered in NPR, the DCR is automatically notified. The register contains date of diagnoses, basis for diagnosis, and tumor characteristics, including ICD-10 diagnosis (since 1978) and ICD-O-3 (the 3^rd^ revision of the International Classification of Diseases for Oncology) codes for morphology and topography, and tumor number.

The *Medical Birth Register* (MBR) was established in 1968 and has been computerized since 1973^27^. Until 1995 data were reported separately to the register, after which the register has received information from the Danish National Patient Register^23^. The register includes data on births and outcomes for all children born in Denmark, including birth weight, malformations, gestational age, and birth complications.

Outcomes ascertained from these registers are outlined in Fig. 3; for HESPERUS chronic diseases will be ascertained in the NPR, DCR and NDR; reproductive health will be ascertained in the NPR and MBR, and neurotoxicity and cognitive function will be ascertained in the DSR (boys and girls) and NCR (only boys ≥18 years).

Persons with incomplete or missing data will be excluded from the statistical analyses. Duplicate data will be cross checked using the unique person number available to all persons living permanently in Denmark. Scientific sound epidemiological research that includes sensitive data such as IQ results is permissible according to Danish legislation after approval by appropriate authorities. Data aggregation and analysis will furthermore be approved by the National Board of Health and the Danish Data Protection Agency before initiation of the study. The study should therefore not include ethical issues.

### HESPERUS

HESPERUS is an example of the first time use of these PCB cohorts and studies a broad range of health outcomes according to level of exposure to lower-chlorinated, semi-volatile PCB congeners in indoor air during different phases of life spanning from conception to middle age and the elderly. These include cognitive function in children, reproductive health and chronic disease (malignant cancer, cardiovascular disease, stroke and type II diabetes) as outlined in Table 4 and were based on previous evidence of health effects associated with the dietary or occupational exposure to higher-chlorinated PCBs.

The possibilities for research using these cohorts are many and could include biological studies based on subsets of the population. For example the reproductive health of sons exposed during gestation could be examined.

## What has been Found?

We have identified two novel cohorts based on indoor exposure to air PCBs: one of pupils attending public schools and one of residents in apartments. These two cohorts, with objective individual health and socioeconomic data from national health and population registries, forms an internationally unique data set to study the impact of exposure to indoor PCBs on human health. The cohorts access hospital, medicine prescription and school grade records in national registers that permit extensive objective follow up for a wide range of diseases and outcomes, and provide epidemiological insights into health effects of indoor air exposure impossible to gain elsewhere^28^. This access to nationwide registers provides a unique and cost-effective option for studying adverse health effects of common exposures.

The cohorts enable scientists to unravel the health effects of indoor air PCB, thus departing from mainstream PCB research that has mainly addressed effects attributable to dietary PCB exposure. The current view is that human toxicity to PCB congeners is primarily driven by non-volatile, dioxin-like congeners in the diet or from occupational exposures. While toxicologists have demonstrated effects of lower-chlorinated, semi-volatile, and mainly non-dioxin-like congeners in experimental models^29^,^30^, there have to date not been feasible options available to study the effects of these congeners in human populations^31^. Moreover, it is well established that non-dioxin-like congeners are the largest contributors for total PCB body burden in humans^31^. With the demonstration that high-level, airborne exposure to low-chlorinated, non-dioxin-like PCB congeners occurs indoors in contaminated buildings, it has now become feasible for us to identify and study a large exposed population. Our method of identifying our cohorts, with complete registration of all students and residents, avoids selection biases which can be present in other observational studies. Furthermore, our linkage with disease registries provides complete disease ascertainment for our cohorts, which is often not possible in other observational studies.

The studies based within these two cohorts will advance current knowledge. Results will enable the reassessment of present action values and lowest tolerable PCB levels for indoor air in non-occupational settings by regulatory bodies such as the World Health organization (WHO) and national health authorities worldwide. If we find no adverse health effects, this could consequently save large amounts of money for PCB remediation expenditures in buildings. On the other hand, if we do find health effects, early disease detection via screening might alternatively save large amounts of money in health care. Finally, the cohorts are based in Denmark which has a population of 5.6 million people living in a small area and exhibiting low emigration rates. However, Denmark is a developed country with a high health standard, and is comparable to all developed countries. Therefore the results of work in these cohorts can be extrapolated to most other countries with similar PCB problems indoors, broadening contemporary understanding of PCB toxicity.

## What are the Main Strengths and Weaknesses?

There are, however, both challenges and limitations: First, the cohorts do not have sufficient sample size to include rare disease outcomes that may be pertinent for PCB air exposure. Nor will they have discriminative power to isolate the effects of different congener types in indoor air, as these are highly correlated within and across individuals. On the other hand, since the level as well as congener profile differs substantially among individuals with and without indoor exposure, we will be able to examine the overall effect of the particular indoor PCB mixture, which differs substantially from the background exposure mainly conferred by the diet. Second, exposure will be estimated based on indoor air concentrations at schools and residences, combined with time spent at these localities, and will entail some misclassification, biasing risk estimates towards zero. Also, in some instances, exposure estimates will be based on air measurements extrapolated from one apartment to another within the same building, which would also imply exposure misclassification. A strength of the study, however, is the prospective design, where outcomes have no possibility of influencing the measure of exposure. Third, although the outcomes selected to date have been carefully selected based upon experimental data, the large number of possible exposure metrics and outcomes make multiple comparisons and chance findings a serious issue. This is to be countered by *a priori* decision on the interpretation of associations – findings demonstrating a linear component to a dose-response relation and consistency among subgroups will be interpreted more strongly. Confounding is also an important issue. For example, exposure as well as cognitive function and school performance are strongly influenced by socio-economic factors. To approach this issue in the school children PCB cohort we will adjust analyses for comprehensive measures of socioeconomic position (mean household income, highest education of parents); confounding in the study of prenatal exposure in the residential PCB cohort will be controlled by design as all children are born within the same housing estate. The possibility of residual confounding by diet or other sources of PCB exposure, though unlikely given the comparability of housing between exposed and unexposed subjects and adjustment for socioeconomic position, cannot be ruled out, and could be evaluated with additional data from targeted sub-analyses, as needed. Finally, although the nationwide registers used in this study are considered to be sound and unique data sources, both the content and the definitions of single variables have changed over time, which may affect validity. Also, the completeness of registrations by health professionals within these registries, and changes in the organization and provision of health services may affect data quality. On the other hand, with the length of follow-up being indefinite, the strength of the study is that long-term follow-up data will be available for scientists in the future.

## How can I Access Data?

In this paper, we have described the birth of two novel Danish PCB-health cohorts of children in elementary schools and residents in apartments who have been exposed to airborne PCBs. Using a strong epidemiological design with robust exposure estimates, these cohorts will enable researchers to unravel potential long term health effects of the lower-chlorinated, semi-volatile PCBs in the indoor environment, offering insight unprecedented worldwide.

Information regarding collaboration on HESPERUS can be obtained by contacting the authors.

## Conclusion

The linking of indoor air measured exposures to PCBs with a number of health outcomes from nationwide registers provides a dataset unprecedented worldwide. HESPERUS will be the first study of the long term health effects of the lower-chlorinated, semi-volatile PCBs in the indoor environment.

## Additional Information

**How to cite this article**: Bräuner, E. V. *et al.* Health Effects of PCBs in Residences and Schools (HESPERUS): PCB–health Cohort Profile. *Sci. Rep.* **6**, 24571; doi: 10.1038/srep24571 (2016).

## Figures and Tables

**Table 1:** Studies of PCB congeners in indoor air.

Area studied	Number of samples	Mean ng/m^3^	Indoor air range ng/m^3^	Sampling period/year	Reference
Farum Midtpunkt Estate, Denmark*	104	1030^1^	ND – 3,843	2011	6
Brøndby Strand Estate, Denmark*	168	1449^2^	ND – 4,750	2011–2015	**
127 schools in Denmark*	342	1097^3^	ND – 3,500	2011–2015	**
Six-school study, USA	64	318£	ND –2,920	2010	32
A contaminated school, USA	96	533	299–1,800	2010–2012	4
A university with known PCB sources, Boston, USA	NR	NR	111 – 393	2001	33
Three PCB contaminated schools, Stuttgart, Germany	19	3889	77–10,655	1994–1995	9
Two schools, Nuremberg, Germany	83	2044£	690–20,800	1999	10
Four classrooms in a highly contaminated school, Germany	4	NR	1,000–12,000	1997	11
One contaminated building in Achen, Germany	65	1740£	ND – 4,280	2004–2005	34
Four public buildings with known PCB sources, Germany	384	NR	715–2,250	2002–2003	35
29 indoor sampling sites, Switzerland	29	NR	720–4,200	2000–2001	36
Recommended action levels for PCB in indoor air		Action to be taken			
Danish recommended action levels***	>3000 ng/m^3^	Immediate need for action to bring levels down			
	300–3000 ng/m^3^	Action plan required to bring levels down			
	<300 ng/m^3^	Exposure not presumed to pose a risk			
USEPA, general population/schools	> = 300 ng/m^3^	Further investigation required			
	<300 ng/m^3^	Acceptable long-term average exposure concentration			

^1^This is the mean for contaminated Farum Estate apartments. Mean for reference Farum apartments: 6 ng/m^3^.

^2^This is the mean for contaminated Brøndby apartments. Mean for reference apartments: 6 ng/m^3^.

^3^This is the mean for contaminated schools. Mean for reference school: 53 ng/m^3^.

^£^This value is a median.

^*^Exposure to be studied in the HESPERUS project.

^**^Unpublished data, obtained within this study.

^***^Recommendations by the Danish Health and Medicines Authority.

NR: Not reported.

**Table 2:** Number of children by air PCB levels^1^ in 127 identified elementary schools.

PCB level (ng/m^3^)/(N_schools_)	<LOD^2^/(26)	1–300/(69)	301–1000/(21)	>1000/(11)
2^nd^ grade 2007–2014				
Total number of children (N = 35,155) Boys (%)	7,620 51.1	18,438 50.7	5,986 51.5	3,111 50.4
Years of attendance (years: mean all children ± SD)	2.16 ± 0.83	2.15 ± 0.84	2.13 ± 0.84	2.15 ± 0.82
9^th^ grade 2007–2014				
Total number of children (N = 31,614) Boys (%)	6,977 51.3	16,261 51.5	5,616 52.0	2,760 51.6
Years of attendance (years: mean all children ± SD)	8.26 ± 2.75	8.24 ± 2.77	8.29 ± 2.72	8.24 ± 2.73

^1^According to PCB air analysis reports; ^2^LOD: Limit of Detection.

**Table 3:** Number of residents of apartments and percentage distribution of total PCB air levels in the social housing estates.

	Farum Midtpunkt Contamination^1^		Brøndby Strand Contamination^1^		All Contamination^1^	
	Yes	No	Yes	No	Yes	No
Residents 1980–2015 (N)	3,482	22,512	2,510	8,681	5,992	31,193
Gender distribution (% male)	50.9	51.3	48.7	51.5	49.9	51.3
Age above 50 years in 2015, %	47.3	43.1	59.8	52.4	52.5	44.2
Duration of stay in dwellings, years, mean	5.3	5.0	7.1	6.5	6.2	5.6
Number of apartments in these housing estates^2^	297	1348	405	480	702	1828

^1^Yes = PCB_total_ in air ≥100 ng/m^3^. Exposure levels in apartments without measurements extrapolated based upon data on PCB levels in building materials, visual inspection, knowledge of building practices in these apartments and location within the housing estate area. With one exception all apartments in an apartment building were contaminated or not contaminated as documented by measurements. Current measures are assumed to reflect past exposure levels from 1970 onwards.

^2^Farum Midtpunkt comprises 27 apartment buildings and Brøndby Strand comprises 12 apartment buildings. Both estates were built during the same time period from 1970–1974.

**Table 4:** HESPERUS design.

Population	Exposure window	Exposure data	Outcomes
Residents in apartments	Adults (n = 37,185)	∑PCB apartment building average, stationary air samples	Malignant disease^2^ Cardiovascular disease^3^ Stroke^3^ Type II Diabetes^3,4^ Reproductive Health^3,5^
Pupils in schools	Children 6–14 years of age (n = 66,969)	∑PCB school average, stationary air samples^1^	Cognitive deficits^6,7^ Reproductive health^3,5^
Offspring of residents	Prenatal and lactational (n = 3,589)	∑PCB and PCB congeners (n = 27), female plasma samples, apartment block average	Cognitive deficits^6,7^

^1^Checked for PCB contamination at the residence.

^2^Ascertained in the Danish Cancer Register.

^3^Ascertained in the Danish National Patient Register.

^4^Ascertained in the Danish National Diabetes Register.

^5^Ascertained in the Danish Medical Birth Register.

^6^Ascertained in the Danish Student Register.

^7^Ascertained in the Danish National Conscription Register.
